# Cod Skin Collagen Peptides Alleviate Ulcerative Colitis by Regulating Gut Microbiota to Inhibit NLRP3 Inflammasome Activation

**DOI:** 10.1002/fsn3.72162

**Published:** 2026-08-07

**Authors:** Longyi Piao, Jiaqi Huo, Yanru Dong, Shuxia Cao, Xiangdan Li, Dongyuan Xu, Xianglan Li, Lan Liu

**Affiliations:** ^1^ Key Laboratory of Cellular Function and Pharmacology of Jilin Province Yanbian University Yanji China; ^2^ Department of Pathology Yanbian University Hospital Yanji Jilin China

**Keywords:** cod skin collagen oligopeptide powder, inflammation, intestinal flora, intestinal mucosal barrier, NLRP3, ulcerative colitis

## Abstract

Enteral nutrition is an important intervention for ulcerative colitis, and collagen can improve immune responses and repair the intestinal barrier. Low‐molecular‐weight peptides from cod skin have demonstrated anti‐inflammatory and mucosal protective effects; however, the mechanism by which they alleviate colitis through gut microbiota modulation remains unclear. This study examined the mechanism by which cod skin collagen oligopeptide powder (CP) affects the gut flora to treat ulcerative colitis (UC). This study evaluated CP in mice with dextran sodium sulfate (DSS)‐induced UC and used 16S rRNA sequencing to analyze its effect on the gut flora. Fecal microbiota transplantation (FMT) demonstrated the role of CP in modulating gut microbiota in the treatment of UC. Hematoxylin and eosin staining, immunohistochemistry, histology, and enzyme‐linked immunosorbent assay (ELISA) assessed CP's effects on colon structure and inflammation. 16S rRNA sequencing showed that CP therapy significantly altered the diversity and organization of the intestinal microbiota in UC mice. FMT demonstrated that CP alleviates UC by modifying the gut microbiota, providing insights into the underlying mechanisms. CP treatment significantly improved body weight, colon length, and histopathological scores in DSS‐induced colitis mice, reduced the levels of NLRP3, Caspase‐1, ASC, IL‐1β, and IL‐18, promoted colonic healing, and alleviated tissue damage. CP inhibited fibrosis by upregulating ZO‐1 and Occludin while downregulating α‐SMA, Vimentin, Snail, and Slug. In conclusion, CP may treat colitis in mice by suppressing NLRP3 inflammasome activation via regulation of the gut microbiota.

## Graphical abstract

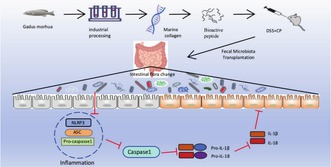

## 1 Introduction

Ulcerative colitis (UC) is a chronic, recurring inflammatory disease of the intestine that typically affects the mucosal layers of the colon and rectum (Ungaro et al. 2017). It is characterized by diarrhea, blood in the stool, stomach pain, and weight loss, and is commonly accompanied by chronic fatigue and anemia (Voelker 2024). UC is caused by several factors, including genetics, immune response abnormalities, microbial dysbiosis, and an unhealthy lifestyle (Adams and Bornemann 2013). Treatment options include aminosalicylic acid, immunosuppressants, and anti‐TNF‐α biologics (Ordás et al. 2012). However, these medications cannot sustain long‐term remission and may cause side effects, including gastric discomfort, nausea, and immunological dysfunction (Di Paolo et al. 2001; Troncone and Monteleone 2017). As a result, it is crucial to develop treatments for UC that are targeted, efficient, and free from toxicity. In recent years, the combination of personalized nutritional interventions and natural bioactive substances has been considered a new trend in the future treatment of UC, providing new perspectives for clinical therapy.

Enteral nutritional support therapy, a new type of intervention, has gradually become an important method for the treatment of UC (Wobith and Weimann 2021). This therapy precisely adjusts the nutritional components of the gut, improves the intestinal microenvironment, promotes the repair of the intestinal mucosa, and restores immune function, thereby effectively reducing inflammation and promoting disease remission. The ocean is the largest reservoir on Earth and is rich in biological resources and medicinal ingredients (Venkatesan et al. 2017). Cod Skin Collagen Oligopeptide Powder (CP), an essential component of marine resources, is a natural collagen isolated from the skin of cod fish that demonstrates the great potential of marine biological resources in the modern health industry (Salvatore et al. 2020; Felician et al. 2018). As a high‐quality collagen oligopeptide, CP is treated by a hydrolysis process, which results in a low molecular weight and enhanced bioavailability (Barati et al. 2020). This oligopeptide powder contains collagen and amino acids, particularly glycine, proline, and hydroxyproline, which have several advantages for the human body (Clark et al. 2008). Studies have shown that cod skin collagen oligopeptide powder has multiple bioactivities including gut health promotion, anti‐inflammatory, antioxidant, and immune system support (Chen et al. 2016; Larder et al. 2023). Studies suggest that CP may decrease the concentrations of pro‐inflammatory mediators like TNF‐α, IL‐1β, and IL‐6, thereby inhibiting inflammatory responses (Cao et al. 2025). It has been used to treat inflammation‐related conditions such as stomach ulcers (Niu et al. 2016), ulcerative lesions (Castro et al. 2007) and for hepatoprotection (Han et al. 2015). Our previous investigation showed that CP ameliorated colitis in mice (Guo et al. 2023). It has also been shown that fish collagen peptides can regulate intestinal flora to ameliorate ulcerative colitis in mice (Yang et al. 2024). However, the mechanism by which CP improves UC by regulating the intestinal microbiota in mice has not been adequately explained.

The NLRP3 inflammasome has been linked to the onset and progression of several illnesses, most notably gastritis and inflammatory bowel disease (Birchenough et al. 2016; Shao et al. 2015). Ulcerative colitis is closely associated with the activation of NLRP3 inflammatory vesicles (Tian et al. 2020). Aberrant activation of the NLRP3 inflammasome not only exacerbates the inflammatory response of the intestinal mucosa, but also creates a vicious cycle of continuous deterioration of inflammation by destroying the intestinal barrier function (Xue et al. 2023). Given this pathological mechanism, selectively blocking the activation of the NLRP3 immune response complex has emerged as a promising new strategy for treating UC. However, the specific relationship between CP and NLRP3 inflammatory remains unclear. The purpose of this study was to determine whether CP alleviates ulcerative colitis by suppressing NLRP3 inflammasome activation via modulation of the gut microbiota. This approach helps clarify the molecular mechanisms of CP in UC management. Therefore, this study aims to investigate whether CP can alleviate ulcerative colitis by regulating the gut microbiota to inhibit the activation of the NLRP3 inflammasome, while also evaluating its potential multi‐target intervention mechanisms. This research will help further elucidate the molecular mechanisms of CP in the treatment of ulcerative colitis and provide a new theoretical foundation for the clinical application of marine bioactive peptides.

## 2 Materials and Methods

### 2.1 Cod Skin Collagen Oligopeptide Powder Preparation Method

Collagen oligopeptides were extracted as follows: First, freeze‐dried or low‐temperature‐dried fish skin was processed to maintain a moisture content of 10%–15%, and impurities, color, and odor were removed through beating, kneading, and air selection. Then, the fish skin was ground into a paste and subjected to heat extraction at 90°C for 1 h. After cooling, the extract was centrifuged to remove the sediment, and the supernatant was collected for enzymatic hydrolysis using Alcalase 2.4 L and Neutrase 0.8 L, with pH adjusted to 7.7 and the temperature maintained at 55°C for 2–4 h. Subsequently, 0.3% activated carbon was added for decolorization and deodorization, followed by centrifugation and filtration after cooling. Finally, the material was concentrated using a vacuum concentrator to below 70°C, and spray‐dried to obtain collagen oligopeptide powder. The process was performed by Yanbian Haijiao Biotechnology Co. Ltd.

### 2.2 Reagents

Dextran sodium sulfate (DSS) (molecular weight: 36–50 kDa) was purchased from MP Bio Medicals (Morgan Irvine, CA, USA). Enzyme‐linked immunosorbent assay (ELISA) kits for mouse TNF‐α, IL‐1β, and IL‐6 were sourced from Mlbio (Shanghai, China). The following antibodies were supplied by Affinity Biosciences (Jiangsu, China): anti‐Snail (#AF6032), anti‐Slug (#DF6202), and anti‐β‐actin (#S0002). Additionally, Affinity Biosciences (Changzhou, China) provided the antibodies anti‐ZO‐1 (#sc‐33,725), anti‐Occludin (#sc‐133256), anti‐α‐SMA (#sc‐53142), anti‐Vimentin (#SC‐7557), anti‐NLRP3 (#DF‐7438), anti‐ASC (#DF‐6304), anti‐Caspase 1 (#sc‐56036), anti‐IL‐1β (#AF‐5103), and anti‐IL‐18 (#DF‐6252).

### 2.3 Animal Experiment and Fecal Microbiota Transplantation Experiment

Male BALB/c mice, aged 6–8 weeks were purchased from the Animal Research Center of Yanbian University. All experimental protocols followed the rules of the China Animal Protection Committee and were authorized by the Institutional Animal Care and Use Committee of Yanbian University. Mice were maintained at 25°C ± 1°C and acclimatized for 1 week prior to the experiment, with appropriate food and water given. The experiments were divided into two phases. Initially, a sum of thirty mice were randomly divided into three groups: control (CON), model (DSS), and CP. Experimental colitis was induced by drinking water in control mice and 3% (w/v) DSS drinking water in the DSS and CP groups. The CP‐treated group received 900 mg/kg by gavage for a test period of 7 days. Next, a total of 40 mice were randomly divided into four groups: CON, DSS, fecal microbiota transplantation (FMT), and CP (*n* = 10 in each group). At the start of the experiment, mice in the control group were given drinking water; experimental colitis was induced by 3% (w/v) DSS drinking water in the DSS, FMT, and CP groups. The CP‐treated group was gavaged with 900 mg/kg, and fresh fecal supernatant from the CP‐treated group was collected and administered to mice in the FMT group by gavage. The experimental period was 7 days. Each mouse in each group was tagged individually and body weights were measured daily. Seven days later, the mice were anesthetized via intraperitoneal injection and fecal and colonic samples were collected. The mice were sacrificed by cervical dislocation at the end of the experiment. Fecal samples were collected from donor mice at the end of the treatment period (i.e., on day 7 of the experiment). Feces from each group of donor mice were weighed and suspended in sterile saline (0.1 g/mL) for homogenization. The resulting fecal suspension was centrifuged at 850*g* for 5 min at 4°C, and the supernatant was collected (concentration > 9.6 × 10^9^ CFU/mL). The collected supernatants were used for FMT and administered to the corresponding recipient mice via oral gavage. The dose of FMT supernatant was 10 mL/kg body weight, administered once daily for seven consecutive days, with each gavage performed as a single administration.

### 2.4 Assessment of Colitis

Each group of animals had their disease activity index (DAI) and histological alterations measured. The DAI score was used to assess inflammation in conjunction with blood in the stool, mucus in feces, and weight loss scores. Mouse colon tissues were collected and treated with 4% paraformaldehyde. The samples were hydrated using an ethanol series followed by soaking in xylene for clearing, embedded in paraffin wax, and sectioned into 4 μm thick slices. Tissue sections were stained with hematoxylin and eosin (H&E), PAS, and Masson's trichrome staining.

### 2.5 Enzyme‐Linked Immunosorbent Assay (ELISA)

Colon tissues were collected, washed with chilled PBS, and subjected to treatment using protease and phosphatase inhibitor solutions. The supernatants were tested for TNF‐α, IL‐1β, and IL‐6 using ELISA kits (mlbio, Shanghai, China), according to the manufacturer's instructions.

### 2.6 Immunohistochemistry

Following the gradient dewaxing process using xylene and ethanol, the colon tissue sections were subjected to antigen retrieval by heating them in a sodium citrate solution at ambient temperature for 15 min. This step aims to restore peroxidase activity. Subsequently, the slices were rinsed thoroughly with PBS, and the washing cycle was repeated three times to ensure proper cleansing. The tissue slices were then exposed to the primary antibody at a 1:300 dilution overnight at 4°C. Afterward, they were thoroughly rinsed three times and then exposed to the conjugated antibody was used for 20 min at ambient temperature. To visualize the results, DAB substrate was used to trigger color development. The samples were clarified, dehydrated, and mounted. Finally, Image J software was used for quantitative analysis.

### 2.7 Western Blot

Proteins from cells and tissues were isolated using the RIPA rapid lysis method. The concentrations of the extracted proteins were measured using a BCA Standard Protein Kit (Thermo Fisher Scientific). Protein samples were separated by SDS‐PAGE and transferred onto a PVDF membrane. The membrane was sealed with skim milk powder for 1.5 h. The primary antibodies, including Snail, Slug, ZO‐1, Occludin, α‐SMA, Vimentin, NLRP3, ASC, Caspase 1, IL‐1β, IL‐18, and β‐actin (diluted 1:1000), were left to incubate overnight at 4°C. Afterward, the samples were treated with the appropriate secondary antibodies for 1.5 h at room temperature to allow for detection.

### 2.8 16S rRNA Gene Sequencing

Fecal samples (3–6 per mouse) were collected and transferred into sterilized 1.5 mL centrifuge tubes and frozen immediately. The samples were stored in liquid nitrogen at −80°C and shipped on dry ice. Total genomic DNA was isolated following the protocol provided with the E.Z.N.A. soil DNA kit. The integrity of the extracted DNA was assessed using 1% agarose gel electrophoresis, while its concentration and purity were verified using a NanoDrop2000. PCR amplification products were identified, purified, and quantified. A sequencing library was prepared using the NEXTFLEX Rapid DNA‐Seq kit, followed by Illumina sequencing on the Miseq PE300 platform. These steps were outsourced to Shanghai Miseq Biotechnology Co., and the resulting data were analyzed using Miseq BioCloud.

### 2.9 Statistical Analyses

The images obtained from the experiments were analyzed using ImageJ software, and the data were statistically processed using GraphPad Prism software. One‐way analysis of variance (ANOVA) was used to compare differences among the treatment groups, followed by Tukey's post hoc test for pairwise comparisons. Paired *t*‐tests were used to evaluate changes in the specific indicators within the same cohort of mice, before and after modeling or treatment. All data are presented as mean ± standard deviation (SD), and *p* < 0.05 were considered statistically significant.

## 3 Results

### 3.1 CP Attenuates DSS‐Induced Ulcerative Colitis

**FIGURE 1:**
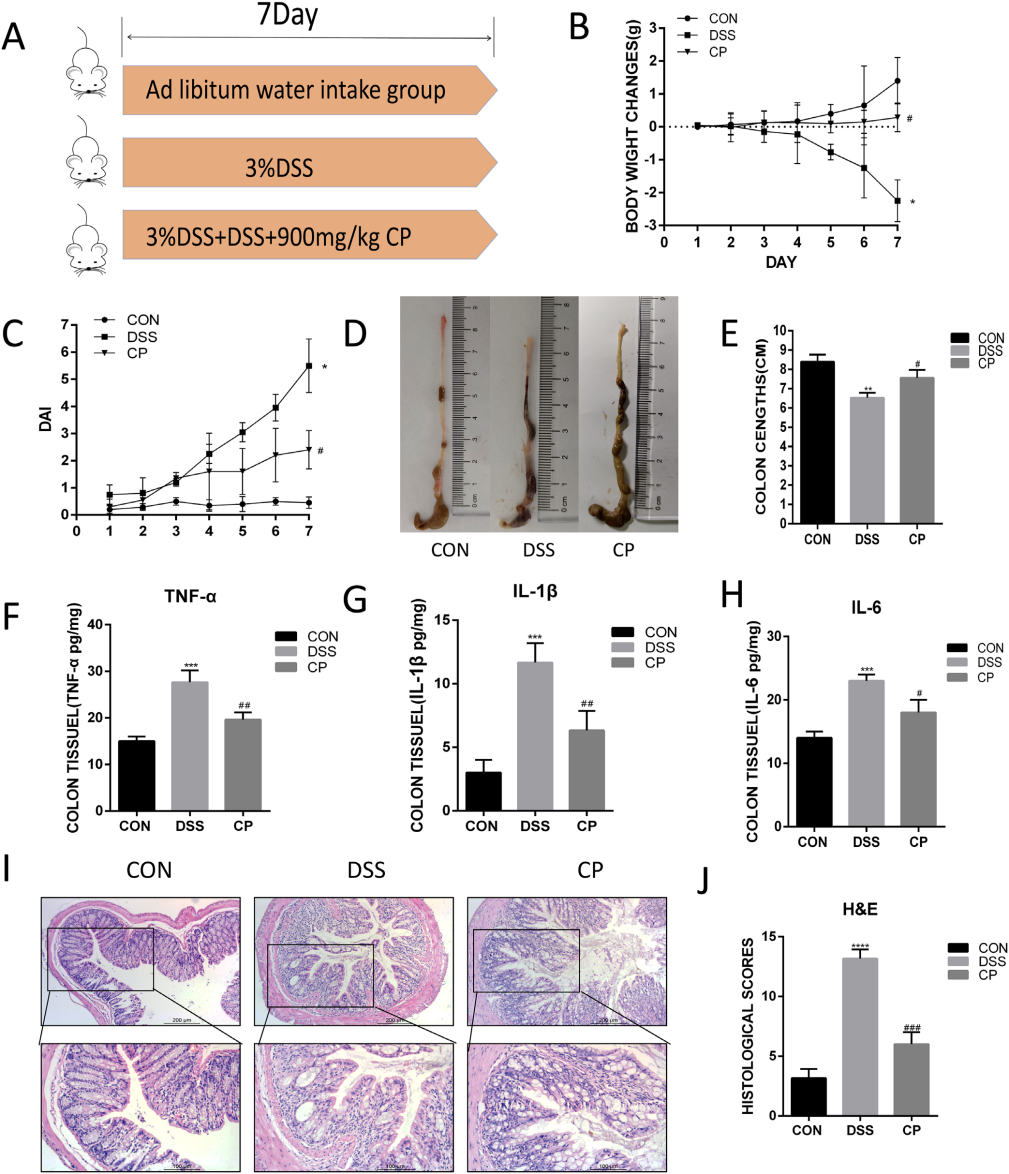
CP attenuates DSS‐induced ulcerative colitis. (A) Experimental design to evaluate the influence of CP on mice induced by DSS. (B) Changes in body weight among the various groups. (C) The evaluation of the extent of disease activity index (DAI) for colitis. (D) The length of the colon in each mouse group. (E) Measurement of the colon length in each group of mice. (F–H) Effects of inflammatory factors in mice. (I) H&E staining showing histological features of the mouse colon. (J) Quantification of H&E staining results. *p < 0.05, **p < 0.01, ***p < 0.001, ****p < 0.0001 versus control group; ^#^ *p* < 0.05, ^##^ *p* < 0.01, ^###^ *p* < 0.001, ^####^ *p* < 0.0001 versus model group. The data in the graphs are provided as mean ± SD. (*n* = 10). Statistical analyses were performed using one‐way ANOVA and *t*‐tests, followed by Tukey's post hoc test.

To test the preventive impact of CP in colitis, a DSS‐induced colitis model was built (Figure 1A). During the trial, we examined the mice's body weights and DAI scores regularly (Figure 1B,C). After 1 week of DSS treatment, mice in the DSS group exhibited characteristic symptoms of colitis, such as considerable weight loss, severe diarrhea, and the presence of blood and pus in their excrement. Additionally, their colons showed considerable shortening and pathological changes (Figure 1D,E). After CP treatment, these symptoms were alleviated, inflammation significantly improved, and the levels of inflammatory cytokines TNF‐α, IL‐1β, and IL‐6 were also significantly reduced (Figure 1F–H). HE staining of colonic tissues showed that the severe intestinal mucosal damage induced by DSS treatment was markedly improved after CP treatment (Figure 1I,J), suggesting that CP could reduce the severity of colitis induced by DSS and reduce colon‐related damage in mice.

### 3.2 CP Increased the Diversity and Abundance of the Gut Microbiota in Mice Induced by DSS

**FIGURE 2:**
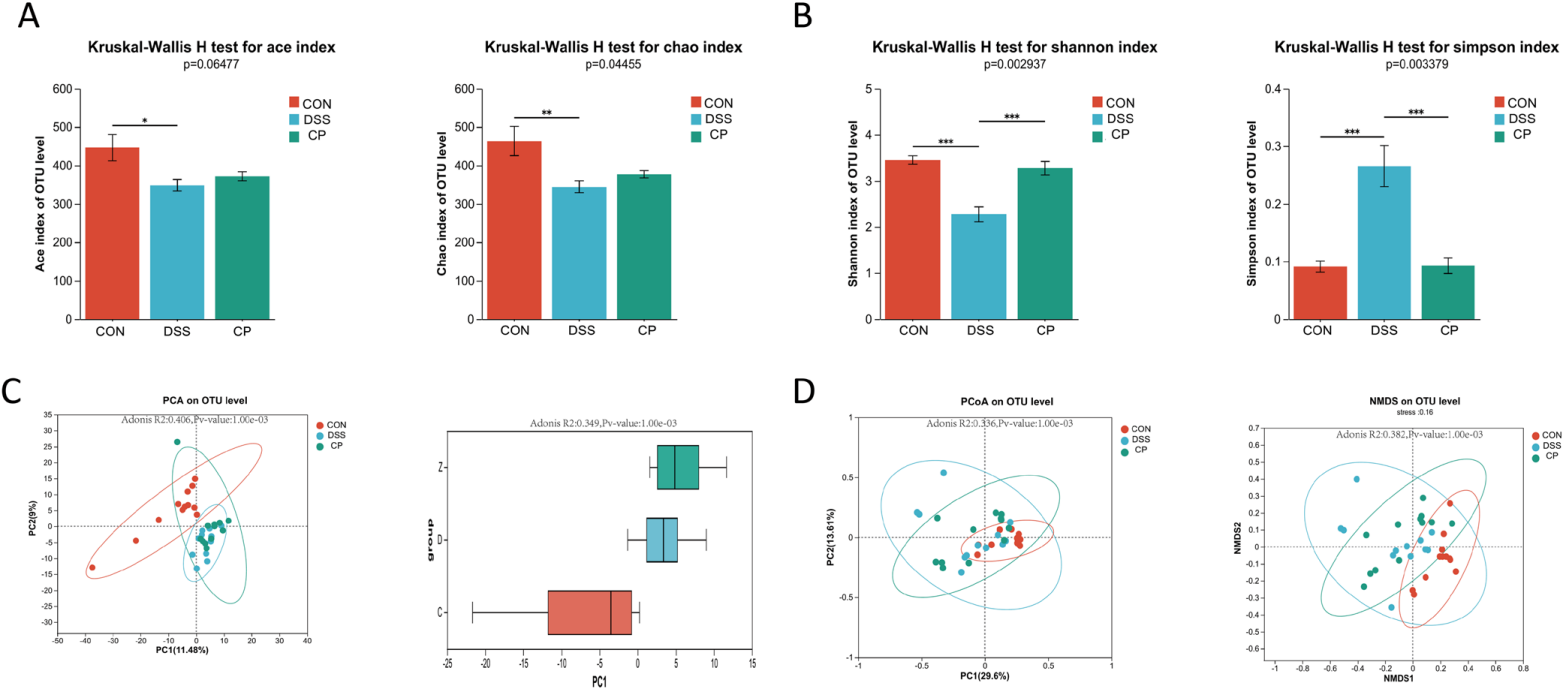
CP increased the diversity and abundance of the gut microbiota in mice induced by DSS. (A) Analysis of α‐diversity using ACE and Chao indices. (B) Analysis of α‐diversity using Shannon and Simpson indices. (C) β‐diversity analysis using PCA. (D) β‐diversity analysis using PCoA and NMDS. *p < 0.05, **p < 0.01, ***p < 0.001, ****p < 0.0001 versus control group; ^#^ *p* < 0.05, ^##^ *p* < 0.01, ^###^ *p* < 0.001, ^####^ *p* < 0.0001 vs. model group. The data in the graphs are provided as mean ± SD (*n* = 6). Statistical analyses were performed using one‐way ANOVA and *t*‐tests, followed by Tukey's post hoc test.

In this study, we utilized high‐throughput sequencing of the 16S rRNA gene to investigate the impact of CP on the gut microbiota. Pairwise Adonis analysis revealed that the gut microbial community significantly differed between the DSS‐induced ulcerative colitis model (MOD) and the control group (CON). Following CP treatment, the microbial community structure did not significantly differ from the CON group, indicating potential restoration of the gut microbiota (Table S1). The DSS group had lower Ace and Chao indices than the CON and CP groups, indicating that DSS treatment reduced the microbial diversity and richness. These indicators were higher in the CP group than in the DSS group, indicating an increase in microbial diversity (Figure 2A). The DSS samples exhibited a notably lower Shannon diversity index, whereas the Simpson diversity index was substantially higher compared with that of NC samples. After CP treatment, the Shannon diversity index of the CP group surpassed that of the model group, although the Simpson's index was markedly reduced (Figure 2B). This indicates that the intestinal microbiota diversity in DSS‐induced UC mice was substantially lower than that in the control group, but the diversity improved after CP treatment in UC mice. To evaluate the similarities and distinctions among samples and groups, we employed β‐diversity analyses, including PCA, PCoA, and NMDS. The findings showed that the CP‐treated group resembled the control group more closely than the model group (Figure 2C,D). This indicated a shift toward restoring the structural balance of the gut microbiota following CP treatment. These results further indicated that CP treatment enhanced the richness and diversity of the gut microbiota by improving its structure.

### 3.3 CP Treatment Alleviated Gut Microbiota Disorders in Mice Induced With DSS

**FIGURE 3:**
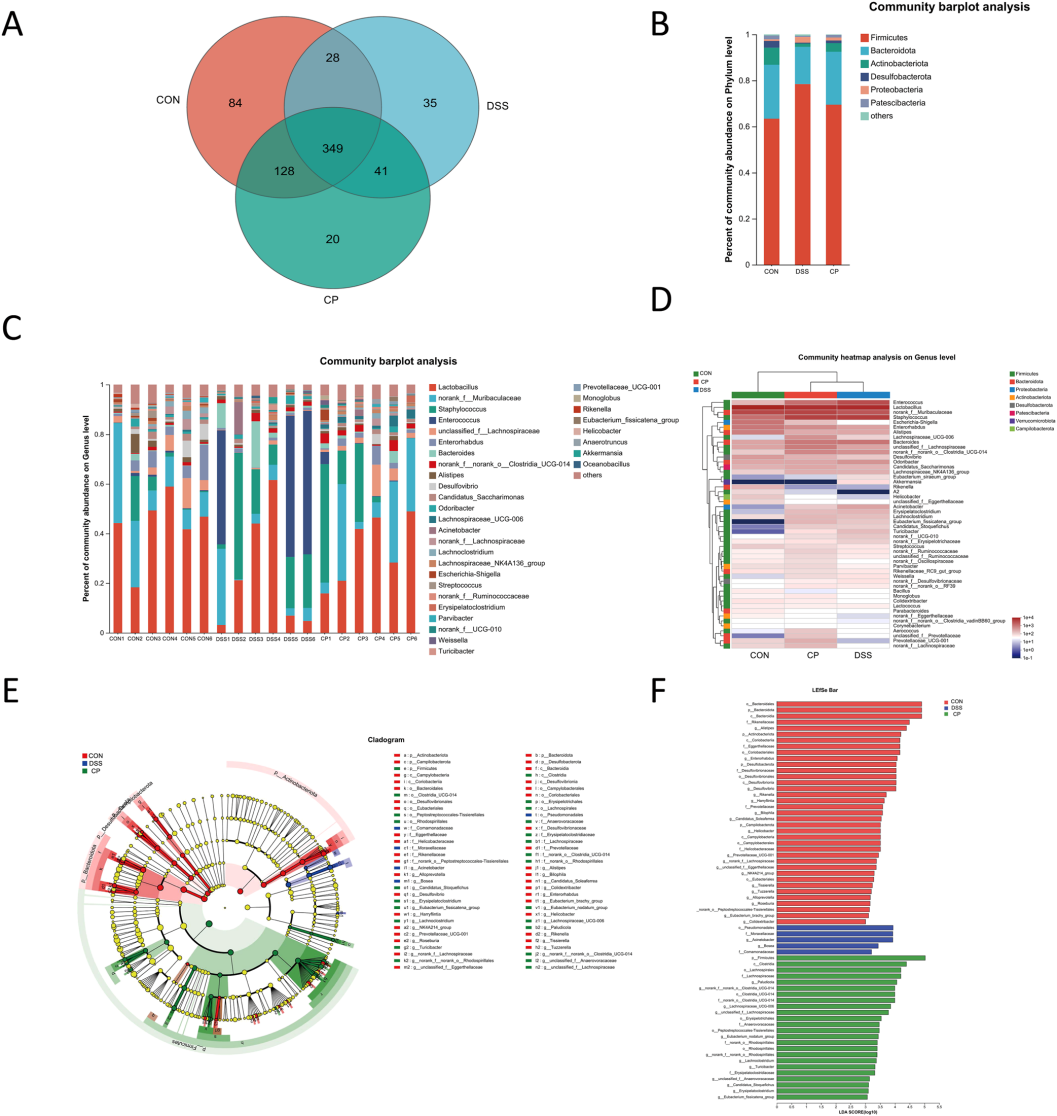
CP administration restored the diversity of intestinal microbiota in mice treated with DSS. (A) A Venn diagram illustrating the composition of different species. (B) The distribution of the intestinal microbiota at the phylum level in each group. (C) Relative abundance of intestinal microbiota at the genus level. (D) Genus‐level heatmap analysis. (E) Cladogram showing LEfSe analysis results. (F) LDA scores of bacterial species that show significant enrichment in each group of gut microbiota (LDA score > 3). The data in the graphs are provided as mean ± SD. Statistical analyses were performed using one‐way ANOVA and *t*‐tests, followed by Tukey's post hoc test.

As shown in Figure 3A, 349 species were shared among the CON, DSS, and CP groups. The CON group has 84 unique species, the DSS group has 35 unique species, and the CP treatment group has 20 unique species. There were 128 species common to both the CON and CP groups and 28 species common to both the CON and DSS groups. These findings demonstrated that the intestinal microbiota in the CP treatment group closely resembled that of the healthy CON group. We analyzed the microbial composition across different groups, focusing on both phylum and genus classifications. At the phylum level, key members such as Firmicutes, Bacteroidetes, and Actinobacteria were identified. Notably, CP treatment led to a marked increase in the populations of Bacteroidetes and Actinobacteria relative to those in the DSS group (Figure 3B). At the genus level, the model group displayed a significant reduction in Lactobacillus abundance, whereas the CP treatment group showed a considerable increase in Lactobacillus abundance relative to the model group. The results showed that DSS treatment significantly reduced the relative abundance of norank‐f‐Muribaculaceae, whereas its abundance was markedly increased following CP treatment. Muribaculaceae is an important commensal group in the gut and is closely associated with polysaccharide degradation and short‐chain fatty acid production. Therefore, the restorative increase in norank‐f‐Muribaculaceae after CP treatment suggests that CP may improve DSS‐induced gut microbiota dysbiosis by promoting the growth of beneficial bacteria. Additionally, the abundance of Staphylococcus and Enterococcus significantly decreased after treatment, further supporting the potential role of CP in restoring the gut microbial balance. These outcomes indicate that CP treatment effectively mitigates the gut microbiota disruption associated with UC (Figure 3C). The species composition heatmap shows the trend of species diversity distribution between the samples and visualizes the compositional differences (Figure 3D). To gauge the effect of CP on the intestinal microbiota profile, the significantly different taxa in the DSS group mainly included Comamonadaceae and Acinetobacter spp. After CP treatment, the number of beneficial bacteria, including Lachnospiraceae, increased. These data suggest that CP treatment helps restore the intestinal microbiota imbalance caused by DSS in mice.

### 3.4 CP‐FMT Relieves Symptoms and Inflammation in DSS‐Induced Ulcerative Colitis

**FIGURE 4:**
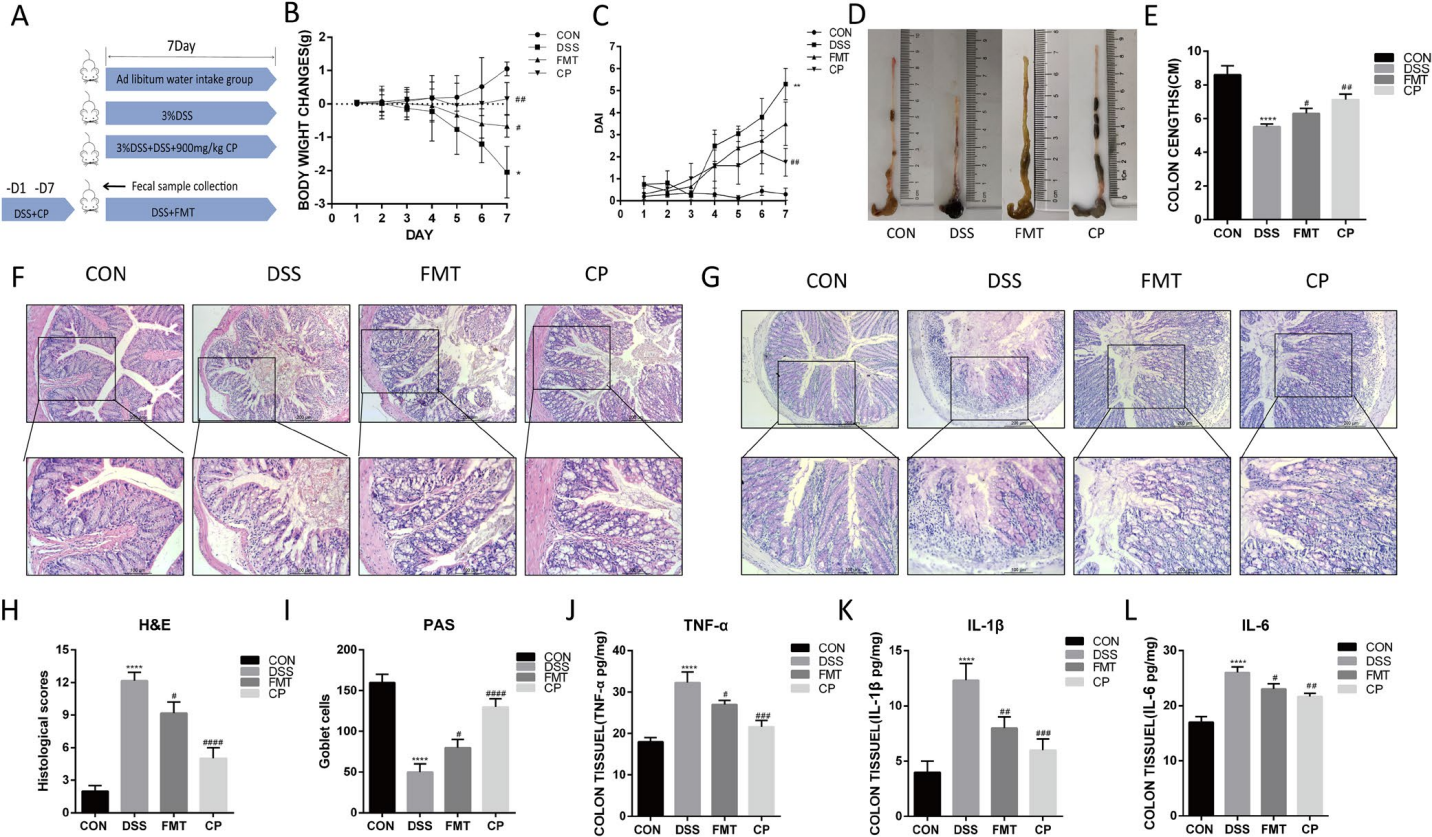
CP‐FMT mitigates the symptoms and inflammation of DSS‐induced ulcerative colitis. (A) FMT experimental design in DSS‐treated mice. (B) Body weight alterations in mice in each experimental group. (C) Appraisal of colitis disease activity index (DAI). (D) Length of colon in each group of mice. (E) Measurement of the colon length in individual groups of mice. (F) Effect of H&E staining to observe the histology of mouse colon. (G) Effect of PAS staining to observe the histology of mouse colon. (H) Quantification of H&E staining results. (I) Quantification of PAS staining results. (J–L) Effects of inflammatory factors in mice. *p < 0.05, **p < 0.01, ***p < 0.001, ****p < 0.0001 versus control group; ^#^ *p* < 0.05, ^##^ *p* < 0.01, ^###^ *p* < 0.001, ^####^ *p* < 0.0001 vs. model group. The data in the graphs are provided as mean ± SD (*n* = 10). Statistical analyses were performed using one‐way ANOVA and *t*‐tests, followed by Tukey's post hoc test.

To further investigate the anti‐inflammatory properties of the gut microbiota in conjunction with CP, an FMT model was developed (Figure 4A). The results revealed that DSS‐induced UC mice experienced a notable decrease in body mass compared with the control group. Nevertheless, CP treatment effectively reversed this decline, with mice in the FMT group showing a marked improvement in body weight relative to those in the DSS group (Figure 4B). As the experiment progressed, the Disease Activity Index (DAI) scores for the DSS model group increased significantly, accompanied by noticeable symptoms of colitis, including diarrhea and blood‐streaked stools. In contrast, both the CP‐treated and FMT groups registered drastically lower DAI scores than the group administered DSS. CP treatment and FMT also improved the shortening of colon length induced by DSS in mice (Figure 4D,E). Hematoxylin and eosin (HE) staining revealed that the colon morphology was intact in one piece in the CON group. In the DSS group, inflammatory cell infiltration severely disrupted the colonic mucosa. In the FMT group, mucosal damage was reduced but inflammation persisted. In the CP group, the colonic structure mostly returned to normal with a substantial decrease in inflammatory cells (Figure 4F,H). The results of Periodic acid‐Schiff (PAS) staining indicated that DSS‐induced colitis mice had a large loss of mucin and goblet cells. However, after FMT and CP treatment, the numbers of both mucin and goblet cells were restored (Figure 4G,I). Additionally, the DSS model group demonstrated substantially increased levels of TNF‐α, IL‐1β, and IL‐6 compared to the CON group. However, pro‐inflammatory cytokine levels decreased markedly following CP therapy and FMT (Figure 4J–L).

### 3.5 CP‐FMT Inhibits the EMT Process to Alleviate DSS‐Induced Colonic Fibrosis

**FIGURE 5:**
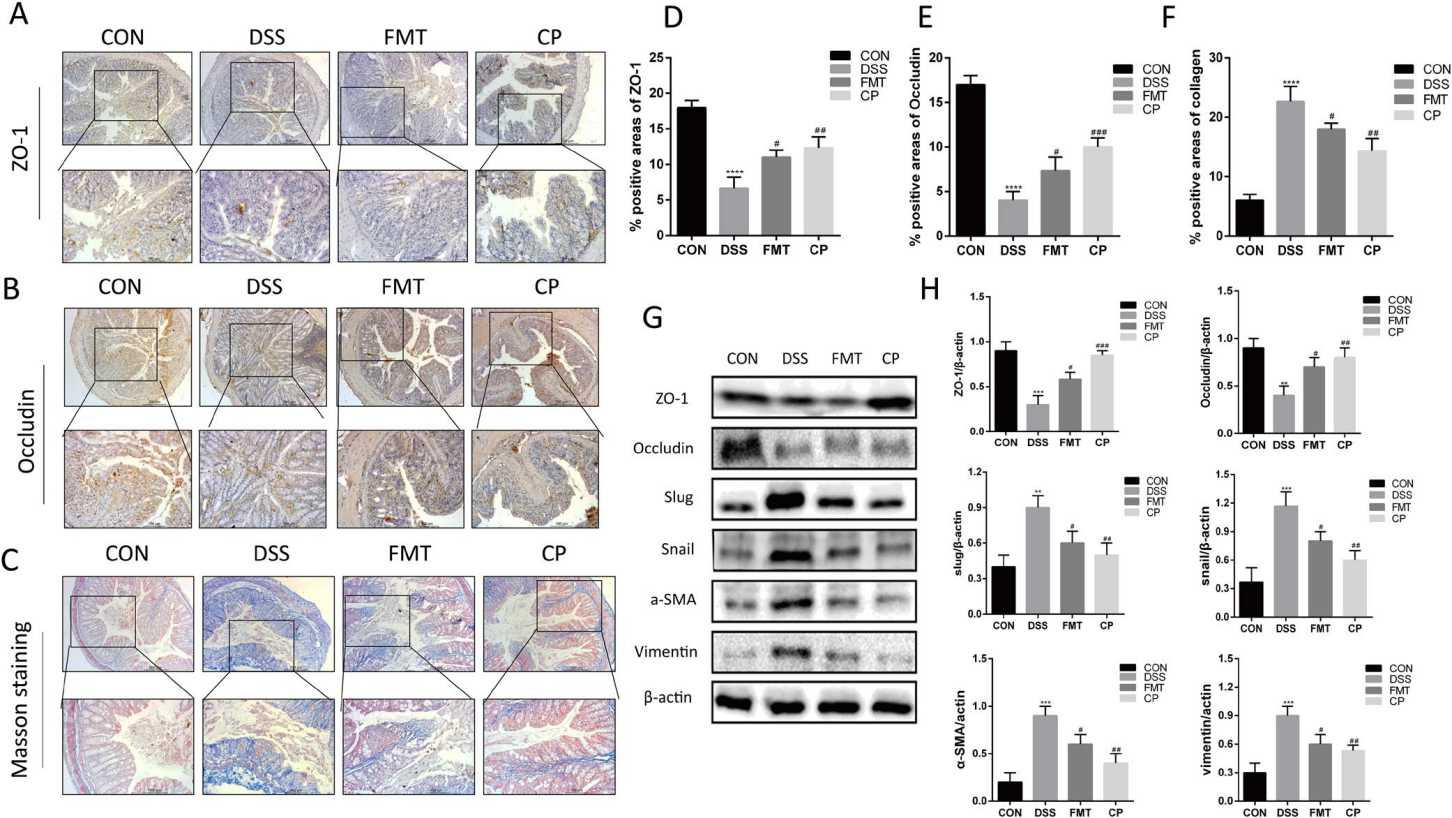
CP‐FMT inhibits the EMT process to alleviate DSS‐induced colonic fibrosis. (A) Immunohistochemical identification of ZO‐1 protein expression in the intestinal mucosa. (B) Immunohistochemical detection of Occludin protein expression in colonic mucosa. (C) Masson staining to observe fibrosis in mouse colon tissue. (D) Quantification of ZO‐1 positive areas. (E) Quantification of Occludin‐positive areas. (F) Quantification of Masson staining of colon tissue. (G) Immunoblot analysis was conducted to examine the occurrence of proteins correlated with epithelial‐mesenchymal transition (EMT). (H) Quantification of EMT‐related protein expression by Western blot. *p < 0.05, **p < 0.01, ***p < 0.001, ****p < 0.0001 versus control group; ^#^ *p* < 0.05, ^##^ *p* < 0.01, ^###^ *p* < 0.001, ^####^ *p* < 0.0001 versus model group. The data in the graphs are provided as mean ± SD. (*n* = 3). Statistical analyses were performed using one‐way ANOVA and t‐tests, followed by Tukey's post hoc test.

EMT is associated with fibrosis and the integrity of the colonic mucosa, leading to increased luminal antigen permeability and mucosal immune dysregulation. Immunohistochemistry showed that DSS disrupted the protective function of the colonic mucosa and decreased the expression of TJ proteins (ZO‐1, Occludin), which was restored by FMT and CP treatment (Figure 5A,B,D,E). Masson staining showed significant collagen accumulation in the lamina propria and submucosa in the DSS group compared to the CON group. This effect was notably ameliorated by FMT and CP treatment (Figure 5C,F). The Western blot findings demonstrate that in the DSS group, the synthesis of tight junction proteins (ZO‐1, Occludin) is diminished, whereas the expression of fibrosis and stromal phenotype proteins (α‐SMA, Vimentin, Snail, Slug) is markedly elevated. Following treatment with FMT and CP, ZO‐1 and Occludin protein expression is upregulated, while the expression of α‐SMA, Vimentin, Snail, and Slug is downregulated, suggesting that FMT and CP can impede the EMT mechanism, uphold the epithelial characteristic and mucosal barrier, and mitigate fibrosis (Figure 5G,H).

### 3.6 CP‐FMT Attenuates DSS‐Induced Ulcerative Colitis via the Suppression of NLRP3 Inflammasome Activation

**FIGURE 6:**
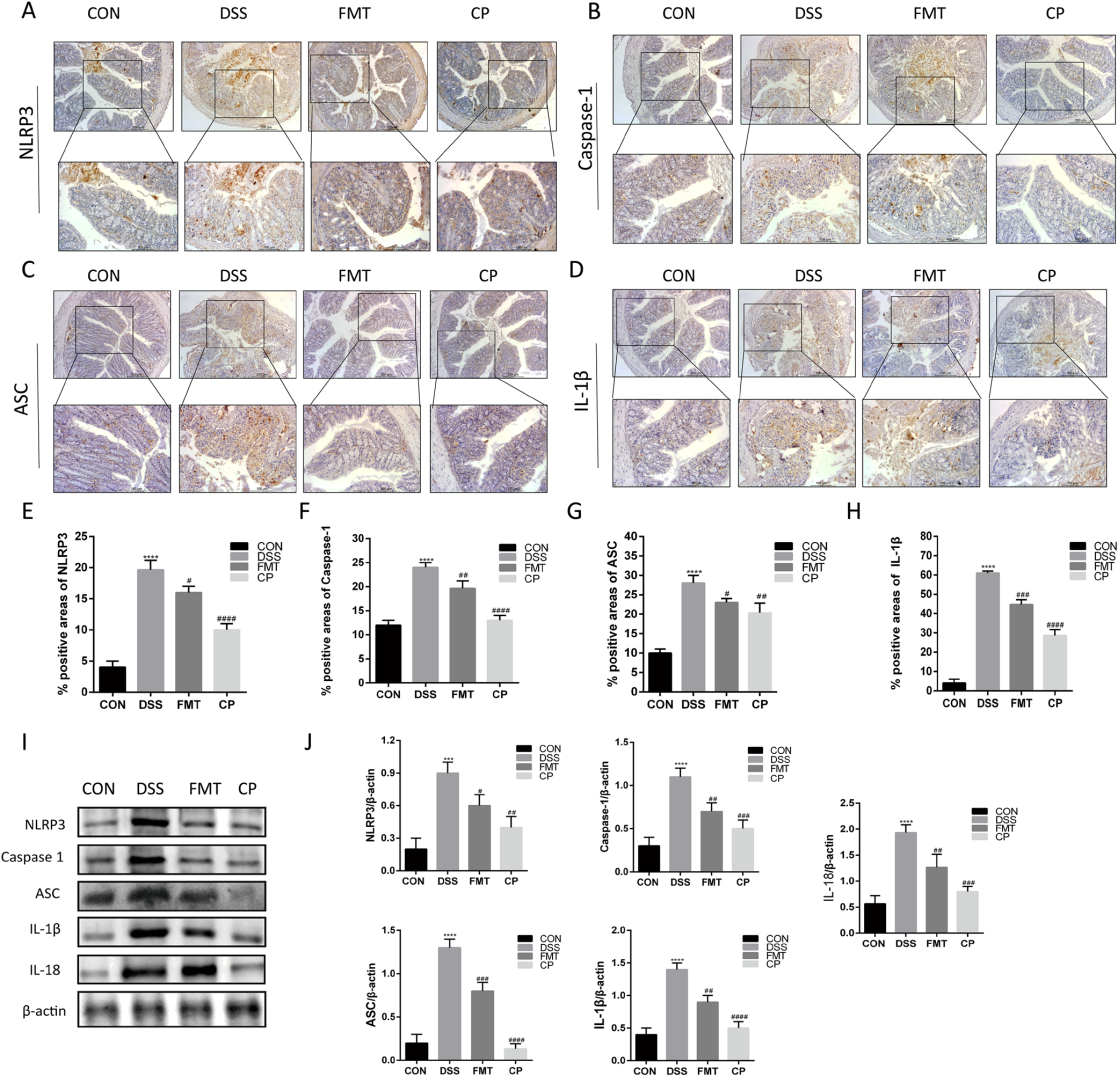
CP‐FMT alleviates DSS‐induced ulcerative colitis by suppressing the activation of the NLRP3 inflammasome. (A–D) Immunohistochemical examination of NLRP3, Caspase‐1, ASC, and IL‐1β protein levels in colon tissues. (E–H) Measurement of the expression quantification levels of NLRP3, Caspase‐1, ASC, and IL‐1β‐related proteins in colon tissue using immunohistochemical staining. (I) Western blot analysis was performed to assess the expression levels of NLRP3, Caspase‐1, ASC, IL‐1β, and IL‐18 proteins in colon tissue samples. (J) Quantification of expression of NLRP3, Caspase‐1, ASC, IL‐1β, and IL‐18 related proteins in colon tissues detected by Western blot. *p < 0.05, **p < 0.01, ***p < 0.001, ****p < 0.0001 versus control group; ^#^ *p* < 0.05, ^##^ *p* < 0.01, ^###^ *p* < 0.001, ^####^ *p* < 0.0001 versus model group. The data in the graphs are provided as mean ± SD. (*n* = 3). Statistical analyses were performed using one‐way ANOVA and *t*‐tests, followed by Tukey's post hoc test.

To further confirm CP's anti‐inflammatory mechanism against DSS‐induced ulcerative colitis, we performed immunohistochemical staining. The results showed that NLRP3‐positive cells were significantly increased and appeared brownish‐yellow in the mucosal and submucosal layers of the DSS‐induced mouse colon, suggesting that an inflammatory response was promoted. This change was significantly more pronounced in the DSS group than in the control group. However, these changes were effectively suppressed in the FMT‐ and CP‐treated groups (Figure 6A,E). Immunohistochemistry findings for Caspase‐1, ASC, and IL‐1β showed a substantial increase in expression within the DSS group when relative to the CON group. However, their expression decreased after CP treatment. At the same time, these proteins also exhibited a downward trend in the FMT group (Figure 6B–D,F–H). Subsequently, we determined the activity of NLRP3 inflammatory vesicle‐associated proteins in the mouse colon tissue using western blotting. The group treated with DSS showed significantly elevated levels of NLRP3, Caspase 1, ASC, IL‐1β, and IL‐18 proteins in their colons (Figure 6I,J). Conversely, the abundance of these proteins was markedly lower in the FMT and CP treatment groups than in the DSS model group, indicating that FMT and CP alleviated DSS‐induced ulcerative colitis in mice by suppressing NLRP3 inflammatory vesicle activation.

## 4 Discussion

Recent studies have shown that marine polypeptides, as substances rich in nutrients and bioactivity, can alleviate ulcerative colitis and enhance immunity by regulating the intestinal immune system and reshaping the gut microbiome (Xiang et al. 2021). Cod skin collagen oligopeptide powder, an important marine biological extract, contains high levels of collagen and its hydrolyzed products and exhibits significant bioactivity (Geahchan et al. 2022). Our results suggest that CP has the potential to reduce inflammation, enhance the resilience of the intestinal barrier, and modulate the gut microbiome. FMT experiments further verified that the CP‐modulated gut microbiota mitigated DSS‐induced UC symptoms by preventing NLRP3 inflammasome activation. Compared with our previous study (Guo et al. 2023), the present study provides novel findings. Specifically, we demonstrated that the therapeutic effects of CP are mediated, at least in part, by microbiota‐dependent modulation of the NLRP3 inflammasome pathway. We also further validated this mechanism using FMT experiments. These findings go beyond the descriptive phenotypic characterization reported in our previous study and establish a causal link between the gut microbiota and the anti‐inflammatory effects of CP. This finding provides new evidence for the application of CP as a novel nutritional adjunct for the prevention and treatment of ulcerative colitis.

The gut microbiota regulates intestinal mucosal function and maintains the equilibrium of the human immune system (Li, Turroni, et al. 2024; Chandrasekaran et al. 2024). An imbalance in the gut microbial community does not only impair the immune defense function of the intestine; it can also trigger chronic inflammation, worsen intestinal pathological processes, and fuel the onset and progression of UC (Wu et al. 2024). Therefore, controlling gut microbiota imbalance is a promising therapeutic method for UC prevention and therapy. In this study, we found a sharp decline in the diversity of the gut microbiota in the DSS group compared to that in the control group, suggesting a gut microbial imbalance in mice. Following CP treatment, the heterogeneity of the gut microbiota was restored. Moreover, the results of PCA and PCoA analyses indicated that CP treatment optimally adjusted gut microbiota disorder in mice. Previous research has demonstrated that the phylum Bacteroidetes contributes to alleviating colitis by interacting in conjunction with the host immune system, metabolizing carbohydrates to produce low‐molecular‐weight fatty acids, retaining the uniformity of the intestinal epithelial layer, and playing a role in systemic metabolism and neurodevelopment (Oliphant et al. 2021). Actinobacteria, including Lactobacillus and Bifidobacterium, are intestinal probiotics that alleviate colitis by modulating the balance of gut bacteria, increasing immunological tolerance, and producing anti‐inflammatory chemicals (Ruiz‐Sánchez et al. 2024). According to some studies, an increase in the abundance of Firmicutes may stimulate the production of mucosal metabolic products, which, in some cases, promote the production of malignant or inflammation‐promoting substances, thereby aggravating intestinal inflammation and leading to microbiota imbalance (Jordan et al. 2023). In this study, at the phylum level, CP administration boosted the prevalence of Bacteroidetes and Actinobacteria while reducing the abundance of Firmicutes. At the genus level, the relative abundance of Lactobacillus increased in the CP treatment group, which is consistent with previous studies. These probiotics facilitate intestinal mucosal barrier regeneration, thus accelerating colon repair (Li, Liu, et al. 2024; Hu et al. 2024).

Currently, reestablishing the equilibrium of the intestinal microbiota is considered a viable technique for addressing ulcerative colitis (Yadegar et al. 2024; Shen et al. 2018). In conclusion, fecal microbial transplantation is becoming increasingly important in the field of studying the interactions between intestinal flora and drug therapy (Li et al. 2022; Chen et al. 2023). FMT is commonly utilized in research for the treatment of colitis (Zhang et al. 2020), and it has been demonstrated that Kaempferol alleviates ulcerative colitis through FMT (Qu et al. 2021). In addition, Banxia Xiexin Decoction significantly improved colitis symptoms via FMT, further validating its efficacy (Luo et al. 2024). In our study, using an UC mouse model, the FMT group demonstrated a significant therapeutic effect compared to the DSS‐treated group. Mice treated with FMT showed marked enhancement in DAI ratings, colon length, histology scores, and goblet cell count. There was also a notable reduction in intestinal inflammation. Simultaneously, we detected a significant increase in the number of goblet cells in both the CP and FMT groups, along with a considerable increase in the expression of Occludin and ZO‐1 proteins. This indicates an improvement in their function regarding the maintenance of barrier function following CP treatment and FMT.

The NLRP3‐related inflammasome, a key pattern recognition receptor in the innate immune system, forms a complex by interacting with ASC, an apoptosis‐associated speck‐like protein, and pro‐caspase‐1 after detecting signals from infections or tissue damage. Activation of caspase‐1 by this assembly promotes the maturation and secretion of pro‐inflammatory cytokines, such as IL‐1β and IL‐18, thereby initiating an inflammatory cascade (Riaz et al. 2021). Studies have demonstrated that the severity of DSS‐induced colitis can be reduced by blocking the triggering of the NLRP3 inflammasome and the synthesis of IL‐1β (Gong et al. 2018). The role of microbial probiotics and their bioactive metabolites, specifically short‐chain fatty acids (SCFAs), in alleviating IgA nephropathy has been demonstrated through the inhibition of the NLRP3/ASC/Caspase 1 signaling pathway (Tan et al. 2022). In the current study, we discovered that CP could control the intestinal immune milieu by remodeling the balance of the intestinal flora, thereby attenuating intestinal pathological irritation. Its mechanism of action involves the inhibition of the initiation of NLRP3 inflammatory vesicles, which in turn reduces the release of pro‐inflammatory mediators and ultimately achieves a protective effect against ulcerative colitis. These findings provide a novel conceptual foundation and therapeutic target for the alleviation of UC. However, this study still has some limitations. For FMT intervention, we used only the fecal microbiota from CP‐treated donors and did not include a DSS‐treated donor as FMT control group. Therefore, we cannot completely rule out the non‐specific effects of the FMT procedure itself, rather than the microbiota‐specific effects, such as stress responses, intestinal irritation, or inflammatory disturbances during transplantation. Future studies should incorporate a DSS‐treated donor FMT control group to more rigorously distinguish the microbiota‐specific effects from the FMT‐procedure effects.

## 5 Conclusions

CP therapy effectively restores intestinal flora equilibrium by lowering the amount of toxic flora while increasing the growth of beneficial bacteria. It inhibits the excessive activation of NLRP3 inflammasomes and the subsequent dispatch of pro‐inflammatory cytokines, thereby decreasing colonic inflammation and improving intestinal barrier integrity. These findings indicate that CP suppresses NLRP3 inflammasome activation through modulation of the gut microbiota, thereby exerting protective effects against ulcerative colitis.

## Author Contributions

**Longyi Piao:** methodology, validation, project administration. **Yanru Dong:** investigation, resources. **Xiangdan Li:** funding acquisition. **Shuxia Cao:** validation, visualization, software. **Jiaqi Huo:** conceptualization, validation, writing – original draft. **Xianglan Li:** formal analysis. **Dongyuan Xu:** conceptualization, writing – review and editing. **Lan Liu:** funding acquisition, supervision, data curation.

## Funding

This study was supported by the National Natural Science Foundation of China (82360479, XD Li) and the Natural Science Research Foundation of Jilin Province for Sciences and Technology (YDZJ202301ZYTS173, Lan Liu).

## Disclosure

All authors stated that this study was not helped by any AI or AI‐related technologies.

## Ethics Statement

The animal study was reviewed and approved by the Animal Ethics Committee of Yanbian University (Ethics No. YD20250122005).

## Conflicts of Interest

The authors declare no conflicts of interest.

## Supporting information


**Table S1:** Adonis analysis.

## Data Availability

The raw datasets presented in the study are stored in the BioProject repository and can be accessed at http://www.ncbi.nlm.nih.gov/bioproject/1246766 under PRJNA1246766.
