# Supplementary material for: Cod Skin Collagen Peptides Alleviate Ulcerative Colitis by Regulating Gut Microbiota to Inhibit NLRP3 Inflammasome Activation
Source: Food Sci Nutr. 2026 Aug 7;14(8):e72162. doi: 10.1002/fsn3.72162 (PMC13451647; doi:10.1002/fsn3.72162)
Supplement: Supplementary file 1 [file FSN3-14-e72162-s001.docx]

**Supplementary Table 1-Adonis analysis**

| **Group** | **Df** | **SumsOfSqs** | **MeanSqs** | **F.Model** | **R2** |
| --- | --- | --- | --- | --- | --- |
| CON_vs_DSS | 1(14) | 0.713(1.716) | 0.713(0.123) | 5.818 | 0.294(0.706) |
| CON_vs_CP | 1(14) | 0.061(2.798) | 0.061(0.200) | 0.304 | 0.021(0.979) |
| DSS_vs_CP | 1(14) | 0.79(1.647) | 0.790(0.118) | 6.719 | 0.324(0.676) |
